# Effects on inequality in life expectancy from a social ecology perspective

**DOI:** 10.1186/s12889-018-5134-1

**Published:** 2018-02-13

**Authors:** Jong In Kim, Gukbin Kim

**Affiliations:** 1 Division of Social Welfare and Health Administration, Wonkwang University, Iksan, Republic of Korea; 2 Institute for Longevity Sciences, Wonkwang University, Iksan, Republic of Korea; 3 Global Management of Natural Resources, University College London (UCL), London, UK

**Keywords:** Inequality in life expectancy, Socio-ecological perspective, Gini coefficient, Secondary education, Labour productivity, Old age pension recipients

## Abstract

**Background:**

Inequality in life expectancy (ILE) is defined as inequality in the distribution of expected span of life-based on data from survival tables estimated using the Atkinson inequality index. ILE can be influenced by socio-ecological indicators including the Gini coefficient, secondary education, output per worker, and old age pension. This study examined the effects on ILE from a social ecology perspective.

**Methods:**

This analysis is based on ILE data from 108 countries obtained from the United Nations Development Programme. Data on socio-ecological indicators were obtained from the United Nations database. The associations between socio-ecological indicators and ILE were assessed using correlation coefficients and multiple regression models.

**Results:**

Significant correlations were evident between ILE and the following indicators from a socio-ecological perspective: Gini coefficient (GC: *r* = 0.335, *p* = 0.001) as an indicator of income inequality, female population with at least some secondary education (FSE: *r* = − 0.757, *p* = 0.001), male population with at least some secondary education (MSE: *r* = − 0.741, *p* = 0.001), output per worker as a measure of labor productivity (OPW: *r* = − 0.714, *p* = 0.001), and number of old age pension recipients (OPR: *r* = − 0.641, *p* = 0.001). In multivariate regression, the ILE predictors were higher GC and lower levels of FSE, MSE, OPW, and OPR (R^2^ = 0.648, *p* < 0.001).

**Conclusions:**

Socio-ecological factors have an important effect on ILE. Policies that address ILE should consider targeted socio-ecological factors, such as the Gini coefficient of income inequality, that give a personal perspective of economic deprivation, attainment of at least a secondary education by both females and males that gives a social environment perspective, output per worker that indicates labor productivity, and the number of old age pension recipients that indicates social security from a public policy perspective.

## Background

We are interested in identifying country-level socio-ecological factors that affect inequality in life expectancy (ILE) among countries. ILE is defined by inequality in the distribution of expected span of life-based on data from life tables estimated using the Atkinson inequality index [1, 2]. The quality of these estimates relies on the quality of the data in the life Table (UN 2016b). ILE can be influenced by country-level socio-ecological indicators that include the Gini coefficient, secondary education, output per worker, and old age pension recipients. Thus, ILE is a useful indicator that can help develop ways to reduce health inequalities [3].

Although studies of ILE have been investigated in various countries [4–7], which socio-ecological factors in various countries affect ILE are unclear [6, 7]. Especially, a retrospective analysis of country-level socio-ecological factors that contribute to ILE could help identify the most important determinants of ILE [3, 8]. With this goal, this study considers how ILE correlates with socio-ecological indicators.

The basic hypothesis of this paper is that as the income level of a country increases, life expectancy increases commensurately [3, 8, 9]. Several studies that estimated ILE for various countries between 2001 and 2012 [4, 6, 7, 10] showed the relationship between black and white populations, social class, indigenous and non-indigenous people, and women and men in terms of ILE. The relationship between income disparity and life expectancy has been reported [9, 11–13], as has the effect of educational inequalities on life expectancy [14–18]. Areas that have been less studied include the relationship of ILE with income inequality, education, labor productivity [19, 20], and the number of old age pension recipients [21, 22].

The present study examines the possible associations between ILE and socio-ecological inequality using several indicators: (1) national income inequality on the personal level [3, 8, 13, 23, 24]; (2) education of at least a secondary education by males and females, which is an indicator of the social environment [15, 17, 18]; (3) labor productivity [19] (Cervellati and Sunde 2005); and (4) the number of old age pension recipients [21], which indicates public policy. The authors expect that countries with low-ILE populations will feature combinations with higher level indicators of socio-ecological perspective (national income, secondary educational attainment, labor productivity, and old age pension recipients).

The determinants of health inequalities are well known. However, ILE is influenced by biological, psychosocial, and environmental factors [3, 8, 18, 24–27]. The aforementioned socio-ecological status components have not been studied in relation to ILE. Life expectancy can be predicted by country-level socio-ecological factors including income inequality and education [9, 11–18, 28], labor productivity, and the population of old age pension recipients [19–22]. Whether these factors are associated with ILE is examined in the present study. In addition, we examine the association between ILE and the GC as country-level personal indicator of income inequality in the whole world, the population of both males and females with at least some secondary education (MSE and FSE, respectively) from a social environmental approach, output per worker (OPW) as a means of indicating labor productivity, and the number of old age pension recipients (OPR) as a measure of public policy perspective. Some studies have investigated the effects of income inequality and schooling on life expectancy. However, the associations between ILE and GC, MSE and FSE, OPW, and OPR have not been examined. This study examined the effects on ILE based on social ecology factors.

## Methods

### ILE framework from a socio-ecological perspective

**Fig. 1:**
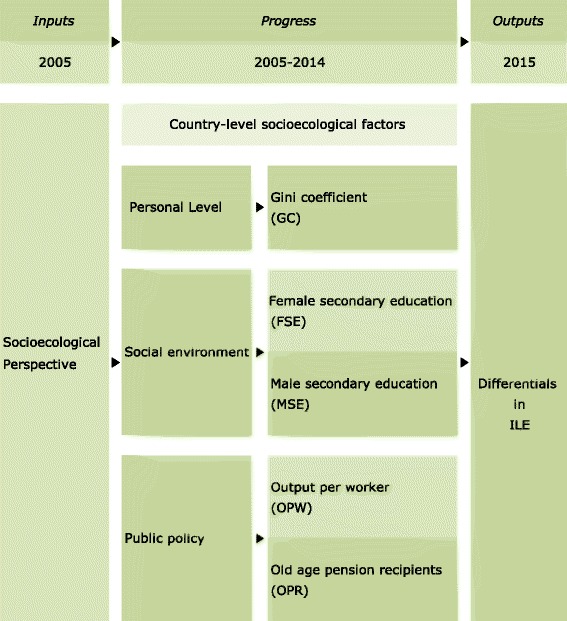
Conceptual framework of country-level socio-ecological indicators for ILE. ILE: Inequality in life expectancy, (%), 2010–2015 . GC: Gini coefficient, (A value of 0 represents absolute equality, a value of 100 absolute), 2005–2013. FSE: Female population with at least some secondary education, (% ages 25 and older), 2005–2014. MSE: Male population with at least some secondary education, (% ages 25 and older), 2005–2014. OPW: Output per worker, (2011 PPP $), 2005–2012. OPR: Old age pension recipients, (% of statutory pension age population), 2004–2012

The framework proposed by this study depicts the socio-ecological indicators of ILE. The relationships between a number of socio-ecological indicators (GC, MSE, FSE, OPW, and OPR) and ILE were examined (Figure 1). Disparities in ILE are influenced by socio-ecological factors. Healthy ageing is a multifactorial characteristic that is influenced by biological, psychosocial, and environmental factors [3, 18, 25–28]. Especially, healthy aging refers to being physically mobile for at least 100 years, or optimising opportunities for good health, without disease, with preserved functional capacity and with a degree of socio-ecological wellbeing, as exemplified through an active life as a part of society [18, 25–33]. However, ILE may be affected or controlled by the socio-ecological environment of a country as well as hereditary factors [28]; the present study excludes ILE-related hereditary or biological factors. This study focused on socio-ecological factors based on a macroscopic theory [3, 18, 26–28].

Based on the collective findings, it is reasonable to think that country-level ILE and socio-ecological indicators may differ between countries (see Figure 1). Some studies have addressed the association between ILE and black and white populations, indigenous and non-indigenous people, and women and men [4, 6, 7, 10].

This study proposes a socio-ecological framework for ILE focusing on interpersonal economic factors, social environment, and public policy as targets for promotion of life expectancy [34–36]. We assume that appropriate changes in the country-level ILE will produce changes in the GC as a measure of economic deprivation from a personal perspective, FSE and MSE attainment from a social environment perspective, OPW as a measure of labor productivity, and the number of OPR as a measure of the welfare of the older population from a public policy perspective.

The hypothesis of this study is that the associations between output (ILE, including differences in ILE between countries) and progress (country-level GC, MSE, FSE, OPW, and OPR) predict health inequalities (Figure 1). These factors can be explained by differences in ILE. Therefore, one is a conceptual model of ILE and the other is a framework comprising socio-ecological indicators that relate to ILE (see Figure 1). In addition, the outputs of this study are based on populations. Thus, the outputs are averaged across the populations and then compared using standard statistical methods (Kim and Kim 2016a, 2017).

### Evaluated ILE

ILE is the inequality in the distribution of expected length of life. It has been used to compare health disparity between countries. Such comparisons inform policy decisions that are contingent on changes in ILE [37]. ILE is defined by inequality in the distribution of expected span of life based on data from life tables estimated using the Atkinson inequality index [1, 2]. Estimates of life expectancy at birth are provided by the United Nations Population Division of the UN Department of Economic and Social Affairs [38, 39]. ILE was calculated from the 2010–2015 life tables from UNDESA in 2015 [38]. This distribution is presented over age intervals (0–1, 1–5, ...., 85+ years), with the mortality rates and average estimated age at death specified for each interval. ILE is estimated from the abridged life Tables (5-year age cohorts) and reflects the current inequality in mortality patterns—some people die before the age of one and others die at 75 or later [40]. The Atkinson inequality index A (1) is calculated as A (1) = 1- (geometric mean length of life / arithmetic mean length of life) [1, 41], but we used data from UN [40], which already applied the Atkinson method, to calculate the ILE (%) from 2010 to 2015.

### Models and statistical methods

Models of this study estimate ILE in relation to each variable have been developed, to examine the associations between differentials in ILE and socio-ecological indicators. The models generate a framework of the components of the socio-ecological perspective [3, 24, 33]. Three models were developed from the socio-ecological perspective. Model 1 considers the (personal level + social environment). Model 2 considers (personal level + public policy). Model 3 considers (personal perspective + social environment + public policy). Predictors of ILE—GC, MSE, FSE, OPW, and OPR—were used to create a model combining the three models. These variables reflect the components of the socio-ecological indicators [3, 24]. The associations between ILE and indicators of socio-ecological perspective in these models are assessed using Pearson correlation coefficients and multiple regression models [3, 24, 28, 33] and assessed the influence in the magnitude of covariates on the ILE.

**Fig. 2:**
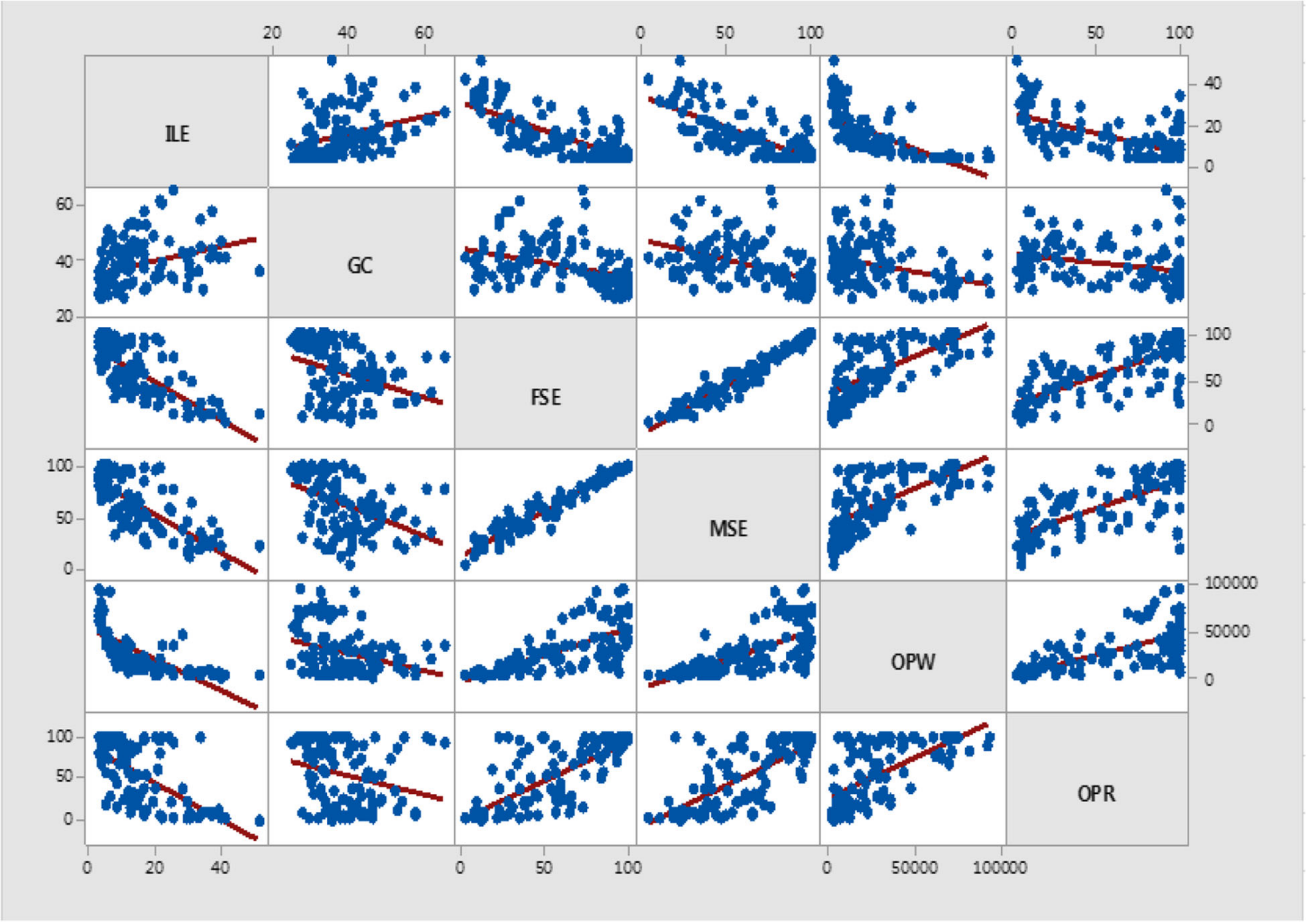
Pairwise scatter plots of all 6 variables; Matrix Plot of ILE, GC, FSE, MSE, OPW, OPR

In addition, the figure with pairwise scatter plots of all 6 variables have with the correlation coefficients in the above diagonal boxes (Figure 2). From the scatters would be able to ascertain whether correlation coefficients are the correct tool to summarise the relationships [42].

### Data collection

Data for ILE analysis were obtained from the ILE study conducted by the UN [2]. The indicators of socio-ecological perspective were obtained from datasets provided by the UN [2, 40, 43]. The study utilized demographic databases from 108 countries.

Six socio-ecological factors were used. ILE from 2010 to 2015, inequality in the distribution of expected length of life based on data from life tables estimated using the Atkinson inequality index, was calculated from the 2010–2015 life tables provided by UNDESA [38, 39]. GC was used; a value of 0 represents utter equality and a value of 100 utter inequality, which presented a measure of the difference in the distribution of income among individuals or households within a country from an entirely equal distribution [2]. FSE from 2005 to 2014 as a percentage of those 25 years of age or older, a percentage of the population aged 25 years or older who have reached a secondary level of education [2]. MSE from 2005 to 2014 which is expressed the same as for FSE. OPW from 2005 to 2012, labor productivity, output per unit of labor input, expressed as GDP per worker, (in 2005 international dollars using purchasing power parity rates, 2011 PPP $), for which data refer to the most recent year available [2]. The sixth factor is OPR from 2004 to 2012, people above the statutory pensionable age receiving an old age pension (contributory, non-contributory or both), expressed as a percentage of the eligible population [43].

## Results

### Differentials in ILE and indicators

Table 1 presents descriptive statistics for the ILE ranges between countries and the values of socio-ecological indicators. ILE ranged from 2.8% in Iceland to 51.2% in Sierra Leone. The mean ILE was 14.82%, with a spread of 48.4%.

**Table 1:** Descriptive Statistics of Variable

Variable	N	Mean	StDev ^a^	Minimum	Maximum
ILE	108	14.82	10.93	2.8	51.2
GC	108	38.12	8.63	24.8	65
FSE	108	58.46	30.74	0.9	100
MSE	108	64.16	27.65	3.2	100
OPW	108	29,829.06	24,751.46	1857	92,694
OPR	108	58.48	37.13	0.9	100

^a^Standard deviation

*ILE*: Inequality in life expectancy, (%), 2010–2015

*GC*: Gini coefficient, (A value of 0 represents absolute equality, a value of 100 absolute), 2005–2013

*FSE*: Female population with at least some secondary education, (% ages 25 and older), 2005–2014

*MSE*: Male population with at least some secondary education, (% ages 25 and older), 2005–2014

*OPW*: Output per worker, (2011 PPP $), 2005–2012

*OPR*: Old age pension recipients, (% of statutory pension age population), 2004–2012

GC ranged from 24.8 in Ukraine to 65 in South Africa, with a mean of 38.12 and a spread of 40.2. FSE ranged from 0.9% in Burkina Faso to 100% in Canada, Austria, Finland, and Estonia, with a mean of 58.46% and a spread of 99.1%. Similarly, MSE ranged from 3.2% in Burkina Faso to 100% in Canada, Austria, Finland, and Estonia, with a mean of 64.16% and a spread of 96.8%.

OPW ranged from $1857 in Malawi to $92,694 in Norway, with a mean of $29,829 and a spread of $90,837. Finally, OPR ranged from 0.9% in Sierra Leone to 100% in Lesotho, Kyrgyzstan, Bolivia, the Maldives, Mongolia, Mauritius, the Russian Federation, Lithuania, Slovakia, the Czech Republic, Finland, Austria, France, Iceland, Sweden, Germany, Netherlands, Denmark, Switzerland, and Norway, with a mean of 58.48% and a spread of 99.1%.

### Predictive variables for the ILE

Data from an analysis of the socio-ecological indicators for the 108 countries are presented in Tables 2, 3, and 4. ILE was correlated with GC, MSE, FSE, OPW, and OPR (Table 2). Significant positive correlations were found between ILE and GC (*r* = 0.335, *p* = 0.001), FSE (*r* = − 0.757, *p* = 0.001), MSE (*r* = − 0.741, *p* = 0.001), OPW (*r* = − 0.714, *p* = 0.001), and OPR (*r* = − 0.614, *p* = 0.001).

**Table 2:** Correlations Coefficient for the ILE

Variables	Correlations Coefficient	*t* -value	*p* -value	R^2^
GC	0.335	3.658	0.001	0.112
FSE	−0.757	−11.937	0.001	0.573
MSE	−0.741	−11.356	0.001	0.549
OPW	−0.714	−10.485	0.001	0.509
OPR	−0.641	−8.598	0.001	0.412

*ILE*: Inequality in life expectancy, (%), 2010–2015

*GC*: Gini coefficient, (A value of 0 represents absolute equality, a value of 100 absolute), 2005–2013

*FSE:* Female population with at least some secondary education, (% ages 25 and older), 2005–2014

*MSE*: Male population with at least some secondary education, (% ages 25 and older), 2005–2014

*OPW*: Output per worker, (2011 PPP $), 2005–2012

*OPR*: Old age pension recipients, (% of statutory pension age population), 2004–2012

**Table 3:** Multiple regression models for predicting ILE

Variables	Coefficient	*t* -value	*p* -value	R^2^
GC	0.174	2.294	0.024	0.439
OPR	−0.594	−7.821	0.001	
FSE	−0.506	−6.351	0.001	0.645
OPW	−0.368	−4.613	0.001	
MSE	−0.479	−6.165	0.001	0.641
OPW	−0.399	−5.141	0.001	
MSE	−0.587	−6.211	0.001	0.569
OPR	−0.211	−2.222	0.001	
OPW	−0.514	−6.191	0.001	0.568
OPR	−0.315	−3.799	0.001	

*ILE*: Inequality in life expectancy, (%), 2010–2015

*GC:* Gini coefficient, (A value of 0 represents absolute equality, a value of 100 absolute), 2005–2013

*FSE:* Female population with at least some secondary education, (% ages 25 and older), 2005–2014

*MSE*: Male population with at least some secondary education, (% ages 25 and older), 2005–2014

*OPW*: Output per worker, (2011 PPP $), 2005–2012

*OPR*: Old age pension recipients, (% of statutory pension age population), 2004–2012

**Table 4:** Multivariate regression models for predicting ILE

Model 1	
Y = 26.302 + 0.074GC - 0.754FSE - 0.024MSE	R^2 ^= 0.578, F-Value =47.459, *P* = 0.001
Model 2	
Y = 21.929 + 0.095GC - 0.491OPW - 0.305OPR	R^2^ = 0.576, F-Value =47.178, *P* = 0.001
Model 3	
Y = 29.092 + 0.031GC - 0.365FSE - 0.098MSE - 0.352OPW - 0.061OPR	R^2^ = 0.648, F-Value =37.651, *P* = 0.001

Model 1: Personal level + Social environment;

(+) Gini coefficient (−) Female secondary education (−) Male secondary education

Model 2: Personal level + Public policy;

(+) Gini coefficient (−) Labour productivity (−) Old age pension

Model 3: Personal level + Social environment + Public policy;

(+) Gini coefficient (−) Female secondary education (−) Male secondary education (−) Labour productivity (−) Old age pension

*ILE*: Inequality in life expectancy, (%), 2010–2015

*GC*: Gini coefficient, (A value of 0 represents absolute equality, a value of 100 absolute), 2005–2013

*FSE*: Female population with at least some secondary education, (% ages 25 and older), 2005–2014

MSE: Male population with at least some secondary education, (% ages 25 and older), 2005–2014

*OPW*: Output per worker, (2011 PPP $), 2005–2012

*OPR*: Old age pension recipients, (% of statutory pension age population), 2004–201

To investigate the direct relationships between ILE and the socio-ecological indicators, multiple regression analysis was conducted. The regression analysis on the socio-ecological indicators revealed the strongest predictors among the three regression models (see Tables 3, 4). Finally, the predictors of ILE were used to build a model in which higher ILE was predicted by higher values of GC and lower MSE, FSE, OPW, and OPR (R^2^ = 0.648, *p* < 0.001).

Lower values of some country-level socio-ecological indicators were associated with a significant effect on the ILE, whereas higher values of GC were also associated with a significant effect on the ILE. These results indicate the great impact of socio-ecological factors on ILE. Thus, countries with low levels of MSE, FSE, OPW, and OPR and high levels of GC had high ILEs, as seen in Model 3.

## Discussion

Shortcomings in socio-ecological perspective are a primary source of inequality. Inequalities in national income, education, labor productivity, and social security have negative ramifications for health promotion development [3, 18, 24, 33]. From a socio-ecological perspective, income inequality (GC), education attainment (MSE and FSE), and labor productivity (OPW) and social security (OPR) have significantly improved over time, but have not led to perfect socio-ecological equity. Socio-ecological inequality remains a major barrier to human development [3, 24, 28, 33]. Inequality from a socio-ecological perspective has increased in many health domains, coinciding with an unequal income distribution [24, 27, 44]. To confirm whether higher ILE is vulnerable to changes in these indicators, this study examined the associations between ILE and socio-ecological indicators.

GC, MSE, FSE, OPW, and OPR contribute to ILE [3, 8, 9, 11–18, 24, 27, 45–47]. Decreases in GC and increases in MSE, FSE, OPW, and OPR lead to decreases in ILE, suggesting that improving these factors can improve ILE.

In the 108 countries examined, MSE, FSE, OPW, and OPR values were the lowest in less developed regions and higher in more developed regions. Country-level socio-ecological factors that influence standard of living, can predict ILE and corresponding country-level socio-ecological inequality [45, 48]. Consequently, socio-ecological indicators are likely major contributing factors to ILE and indirectly reflect country-level socio-ecological conditions required for healthy living. Therefore, as individuals’ health status and standard of living apparent based on socio-ecological indicators values decrease and increase, so too does ILE. The national income inequality index, secondary education attainment, labor productivity, and social security are controlled measures that are crucial determinants of ILE.

In addition, higher levels of income inequality and relative poverty occur in more developed countries [23, 24, 27, 49]. However, presently more developed countries displayed higher income and educational status, and lower ILE. In less developed countries, country-level income and educational inequality have likely contributed to poor progress in attaining health equality [18, 24, 27]. During the study period, as income inequality determined by the Gini coefficient decreased and education level (at least secondary education) increased, ILE decreased as well. ILE had a consistent influence, independent of income inequality and secondary education attainment from a socio-ecological perspective.

Access to labor productivity and social security via OPW and OPR can improve the quality of life and authorize individuals to take charge of their own health, which ultimately promotes better health [18, 33]. Raising the age at which senior citizens qualify for public pension benefits is a detriment to the health of low income seniors [50]. Life expectancy appears to be a real labor productivity effect [51]. Better health increases the labor supply and productivity. Historically, advances in health have been major contributors to economic growth [52]. Thus, OPW and OPR can be seen as innovative tools for health promotion when viewed from a public policy prospective, and their values have implications that can help to improve the standard of living.

A limitation of this study is a lack of comparable data between some of the countries. The inequality-adjusted human development index in the UN database [40] captures inequality using the index of human development. However, it is not association-sensitive, meaning that it does not account for overlapping inequalities (whether the same people are at the lower end of each distribution). In addition, our findings may not apply to individuals in a particular population, which is a limitation of a socio-ecological study. However, this problem also applies to other observational studies and randomized controlled trials [24, 27]. This study would not be able to the selection of education variables that other education like primary, higher and literacy status.

Finally, our hypothesis could be tested that the associations between ILE and country-level socio-ecological factors predict the years of longevity and full health of a population. In the proposed models, it is evident that if countries improve values of GC, MSE, FSE, OPW, and OPR, they can obtain lower ILE. Therefore, policies that improve these country-level socio-ecological factors are expected to have latent effects on ILE. In addition, a lesson from the experiences of the 108 countries studied is that governments should attempt to reduce income inequality and increase access to education in secondary education, labor productivity, and access to old age pensions. Meeting these goals could decrease the risk factors for ILE and increase the standard of living. Thus, the findings of this study must be used to implement strategies related to ILE. These strategies should include addressing country-level socio-ecological indicators.

## Conclusions

This study identified five country-level socio-ecological indicators as important contributors to inequality in life expectancy. These were higher overall national income inequality level, lower female and male secondary education attainment, lower labor productivity, and fewer old age pension recipients. Country-level socio-ecological indicators seem to have an important effect on inequality in life expectancy. Thus, policies that address country-level inequality in life expectancy should consider target socio-ecological factors, such as the GC of income inequality, as an intrapersonal measure of economic deprivation, female and male attainment of at least a secondary education from a social environment perspective, output per worker as labor productivity, and the number of old age pension recipients from a public policy perspective.

## Abbreviations

FSE: Female population with at least some secondary education
GC: Gini coefficient
ILE: Inequality in life expectancy
MSE: Male population with at least some secondary education
OPR: Old age pension recipients
OPW: Output per worker (labour productivity)
